# Psychosocial needs of post-radiotherapy cancer survivors and their direct caregivers – a systematic review

**DOI:** 10.3389/fonc.2023.1246844

**Published:** 2023-10-26

**Authors:** Ka Hei Man, Helen Ka-Wai Law, Shing Yau Tam

**Affiliations:** ^1^ Department of Health Technology and Informatics, Faculty of Health and Social Sciences, The Hong Kong Polytechnic University, Kowloon, Hong Kong, Hong Kong, SAR China; ^2^ School of Medical and Health Sciences, Tung Wah College, Kowloon, Hong Kong, Hong Kong, SAR China

**Keywords:** cancer, cancer survivors, caregivers, psychosocial needs, radiotherapy

## Abstract

Radiotherapy is an important modality for cancer treatment. About 50% of cancer patients receive radiotherapy, and one-third of radiotherapy recipients were identified as having unmet psychosocial needs. The unmet psychosocial needs worsen the patient’s quality of life and treatment effectiveness. This review aims to identify the psychosocial needs of post-radiotherapy cancer survivors and their direct caregivers. Systematic research of Embase, Scopus and PubMed was done and 17 studies were selected for analysis. The results show that patients encounter distress and fear due to treatment immobilization and unfamiliarity with procedures respectively. Information provision is a common need raised by patients and caregivers. Patients and caregivers report relationship problems due to affected sexual functions. To facilitate future studies, solutions to each identified psychosocial need are proposed in the discussion based on the 17 selected papers and other supporting literature. This review proposes art therapy to alleviate psychological distress, and pre-treatment information sessions to reinforce information delivery. Creative interventions such as a sexual rehabilitation program are recommended. Future studies are warranted to examine the interventions and thus improve the patients’ and caregivers’ well-being.

## 1 Introduction

In recent years, there has seen remarkable progress in cancer treatment and development. However, cancer remains a global burden. In 2020, more than 18 million cancer cases were diagnosed worldwide (1). Radiotherapy is one of the most common and important cancer treatment modalities. It has been estimated that approximately 50% of all cancer patients receive radiotherapy during their course of illness (2). This high prevalence of radiotherapy applications signifies the importance of conducting radiotherapy-related studies to improve treatment effectiveness and patients’ quality of life.

Radiotherapy uses high-energy radiation such as X-rays and gamma rays to destroy or damage cancer cells (3). Unavoidably, radiotherapy will cause side effects that affect patients physically and mentally. Physically, normal and healthy cells near the tumor cells are damaged by radiation, leading to mutation or complications. There are some known cancer-specific physical side effects according to the irradiated sites. For instance, abdominal cramps and watery diarrhea are commonly seen due to acute radiation toxicity in the gastrointestinal tract (4). Another example is the damage to the salivary gland during head and neck cancer radiotherapy which may lead to cell death, causing swelling and tenderness after treatment (5). Eating and speech difficulties may arise subsequently. As full recovery of salivary gland function takes months or years, post-radiotherapy patients’ quality of life can be significantly affected.

At the same time, radiotherapy may also deteriorate patients’ mental health. A recent study shows that affective psychological comorbidities greatly influence radiotherapy patients’ quality of life (6). Receiving radiation to treat cancer is likely to increase the risks of anxiety and depression, and lowers patients’ psychological and social well-being. It has been reported that up to 49% of patients attending radiotherapy appointments experience anxiety and mental distress (7). There are cancer-specific psychosocial needs. For instance, rectal cancer patients may suffer from fatigue or sleepiness due to nocturia (8). Due to decreased sexual functioning, gynecological cancer patients may suffer from relationship and social problems (9). Additionally, cancer patients are usually taken care of by caregivers such as their family members and friends. In this review, the targeted caregiver is a layperson instead of a professional caretaker. The caregivers’ mental health may also be affected due to long-term care for the patients. Therefore, cancer is described as a serious and chronic disease affecting patients and caregivers, showing the need for psychological treatment and related studies (6).

Psychosocial needs refer to the combination of mental health, emotional, spiritual or behavioral needs and concerns which are important to the patient (10). Common psychosocial needs include the provision of information and communication, emotional support and family involvement. Low psychological well-being and a lack of social support are commonly reported in radiotherapy patients (11). Up to one-third of patients treated with radiotherapy have been identified as having unmet psychosocial needs (7). There is a gap between the existing healthcare services and the care that the patients and caregivers want. Most importantly, these unmet needs may result in refusal to receive radiotherapy, treatment delays and low adherence to medical advice (6). Therefore, we believe that addressing the psychosocial needs of patients and caregivers can improve the quality of care service provided. The current loophole explains the importance of investigating the psychosocial needs of the patients and caregivers in order to better address their demands.

Notably, few studies have investigated the psychosocial needs of post-radiotherapy patients and their caregivers. There is a wide variety of needs and limited overviews of summarization in this field. A study shows that the lack of attention to psychosocial needs may be attributed to a lack of skills or available interventions (12). Therefore, this review aims to identify the psychosocial needs of post-radiotherapy patients and their direct caregivers. To facilitate future research, interventions tackling the identified needs are suggested in the discussion section. The solutions are proposed based on literature and are not necessarily described in the 17 selected papers for content analysis.

## 2 Materials and methods

### 2.1 Data source

This systematic review was performed according to the Preferred Reporting Items for Systematic Reviews and Meta-Analyses (PRISMA) recommendations (13). This research employed 3 online databases including Embase, PubMed and Scopus. The study title was designed before the literature review process. During the online research process, the title was further amended and finalized.

After finalizing the study title, an online literature search was conducted in late 2022 with the aid of the advanced search function. The advanced search included inputting keywords (“psychosocial needs” OR “psychological needs” OR “mental needs”) AND (“radiotherapy” OR “radiation therapy”) AND (“patients” OR “caregivers”). These keywords or their synonyms must appear in the title or abstract.

Major themes concerning psychosocial needs were identified across articles based on content analysis of their findings. These themes are further examined in the discussion section with possible solutions proposed. For the literature review, only English sources with full texts were selected. To obtain updated information for review, only the articles published within the last 5 years (2017–2022) were selected. Since cancer is a global problem, no filter was placed on the place of publication. Additionally, only primary studies were considered and all reviews were excluded. To ensure constructive findings and generalizability, case reports were not considered. Due to the very limited number of papers focusing on some particular cancer types such as colorectal cancer, this review investigated the general post-radiotherapy psychosocial needs without specifying the cancer type.

### 2.2 Data extraction and exclusion

After identifying the available papers, the title and abstract of each paper were examined to study their relevancy and eligibility. Articles were excluded based on three reasons. Firstly, this research focused on cancer patients’ psychological and social needs. Therefore, articles related to physical side effects or needs were classified as irrelevant to this review and were not included.

Secondly, this review only focused on the psychosocial needs of post-radiotherapy patients and caregivers. Some studies determined the needs of cancer patients after surgery or chemotherapy. Therefore, their findings were irrelevant to this review.

Other than the non-radiotherapy modality issue, papers studying multiple treatments were excluded. Some articles reported the patients’ needs on more than one treatment modality such as radiotherapy and surgery. They were excluded as it was impossible to isolate the psychosocial needs which were specifically caused by radiotherapy. This review aimed to identify the psychosocial needs commonly encountered by post-radiotherapy patients and their caregivers, which was a less studied topic, so studies were restricted to radiotherapy only.

Thirdly, the targets of this review were cancer patients and their caregivers. Some papers were excluded due to the unsuitable target. For instance, journals related to the needs of medical professionals were excluded.

Other than these three common reasons, there were still some common features of the excluded papers. For example, single case reports were not selected for this research due to their low generalizability. Before categorizing the common features and analyzing the findings, the results of each article were read to ensure relevant and factual conclusions. Inclusion and exclusion criteria are summarized in **Table 1**.

**Table 1:** Inclusion and exclusion criteria of the article selection process.

Inclusion criteria	
1. Original research articles
2. Articles published within the last 5 years (2017–2022)
3. All publication places
4. English articles with full texts
5. All cancer types (Specific or non-specific)
Exclusion criteria	
1. Articles studying physical side effects or needs
2. Not studying effects of radiotherapy
3. Articles studying multiple treatment modalities
4. Articles not studying the needs of patients and/or caregivers
5. Case reports or review articles

### 2.3 Quality assessment and data analysis

To compare the quality of the selected papers, the methodological index for non-randomized studies (MINORS) was used as the grading approach (14). There are eight grading criteria looking into the aims, inclusion of patients, data collection, endpoints and follow-up period, unbiased assessment, loss of follow-up and study size. This review involved a general characterization of major design features of studies such as the eight criteria mentioned. Also, major thematic issues were investigated with proposed solutions.

If a paper fully satisfies a criterion, two marks are given. If a paper slightly fulfills the requirement, one point is given. A zero mark is scored for papers which fail to meet the criterion. The maximum score is 16. The papers with 10 points or above are classified as “good”, and those below 10 points are classified as “bad”.

## 3 Results

### 3.1 Study research

**Figure 1:**
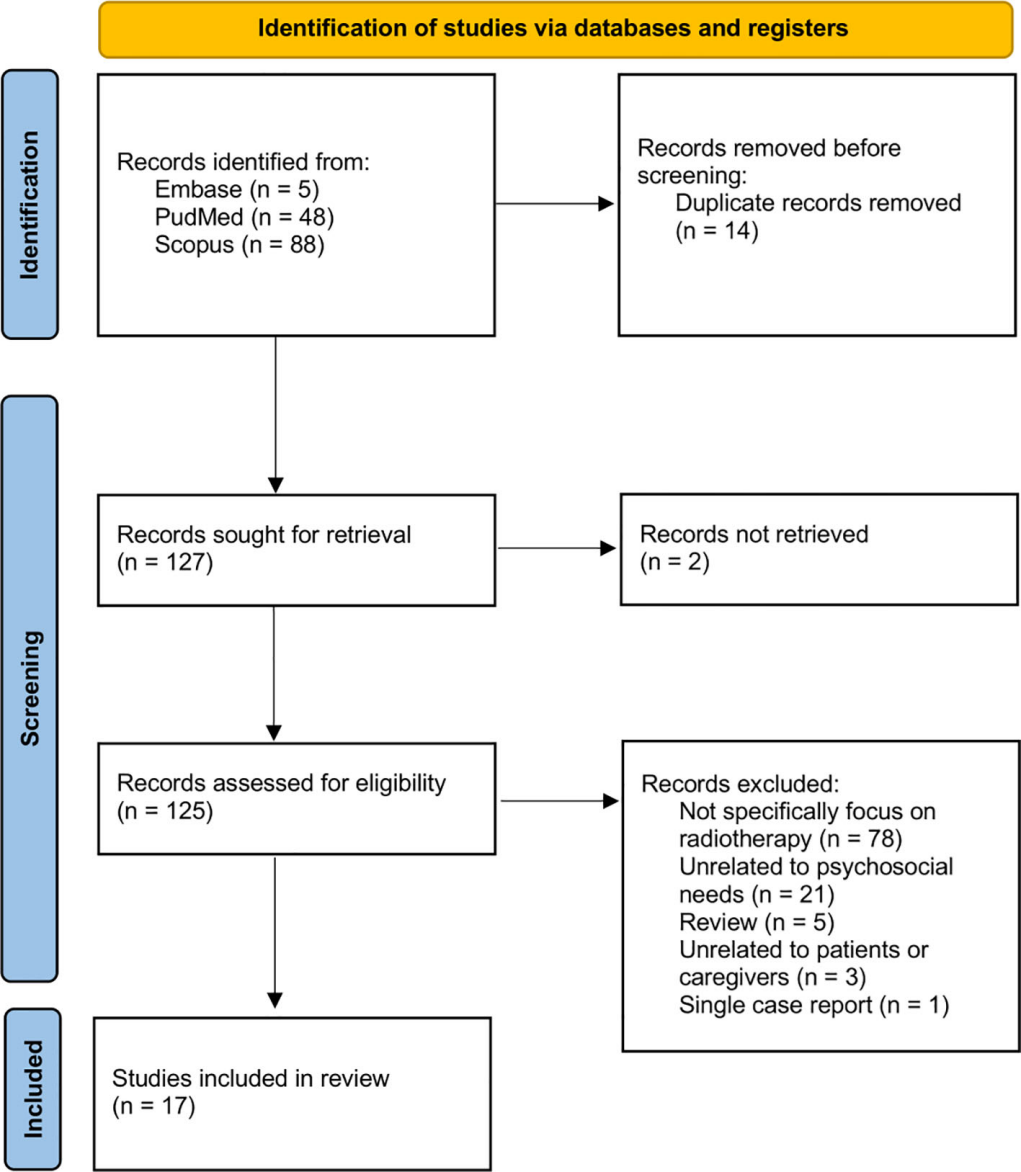
PRISMA flow diagram illustrating the date selection and screening process (13).

The data extraction was conducted by one single investigator (K.H.M.) The literature search resulted in 127 articles after removing duplicate records. In total, 125 publications were sought for retrieval with full texts. Next, all 125 papers underwent full review, and 17 were included in the final analysis. Seventy-eight papers were excluded as they did not specifically focus on radiotherapy, being the most significant reason for exclusion. Studies that only investigated radiotherapy and the derived psychosocial needs were included for analysis and discussion. The second most significant exclusion criterion was being unrelated to psychosocial needs. Twenty-one articles were excluded, most of which solely investigated physical needs such as prophylactic tooth extractions (15). Only independent studies but not reviews nor case reports were included in the 17 articles selected for final analysis. The PRISMA flow diagram in **Figure 1** illustrated the literature review and screening processes.

### 3.2 Study characteristics

Among the 17 articles, the studies on the psychosocial needs of patients are more prevalent than that of caregivers. Nine out of 17 papers investigated the psychosocial needs of post-radiotherapy cancer patients (6, 12, 16–22). 7 papers investigated both patients’ and caregivers’ needs (9, 11, 23–27). Only 1 article investigated the needs from the caregiver’s perspective only (28). Generally, patients are usually the primary target of the study. Meanwhile, the caregiver’s needs are also discussed together with the patient’s needs such as the psychological impacts on families and friends, and the emotional detachment of the patients’ partners (25, 26).

There were 3 randomized controlled trials (9, 12, 24). Others were observational studies. For the type of cancer investigated, 3 out of 17 papers study breast cancer (6, 18, 25). There were 3 papers investigating head and neck cancer (12, 16, 23) and gynecological cancers respectively (9, 19, 21), being the most commonly studied cancer types in this review. Five out of 17 papers had no specified cancer type, focusing on a range of cancer treated by radiotherapy (11, 17, 20, 24, 27). For instance, there was a study that investigated cancer ranging from urological tumor to pancreatic cancer (11). Notably, all the articles investigated radiotherapy as the only treatment. The characteristics of the selected papers were listed in **Table 2**.

**Table 2:** The characteristics of the 17 selected papers include disease investigated, subject number, intervention investigated, and tool of measurement.

Article	Disease investigated	Subject number	Targets investigated	Intervention investigated	Tool of measurement	Key findings
Adeberg et al. (16)	Head and neck cancer	1020	Patient	Psycho-oncological care program	Questionnaires	A
Ahmadsei et al. (17)	Various cancers	33	Patient	Not mentioned	Questionnaires from EORTC	A
Andersen et al. (18)	Breast cancer	7	Patient	Not mentioned	Interviews	B
Ashmore et al. (19)	Gynecological cancer	10	Patient	Digital health intervention	Voice recording	B
Braat et al. (12)	Head and neck cancer	55	Patient	Nurse-led aftercare intervention	Conversation	Non-specific
Catherine et al. (6)	Breast cancer	47	Patient	Affective psychopathology	Interviews	A
Chiesa et al. (20)	Various cancers	99	Patient	A multidimensional assessment tool (M.A.P.-RT schedule)	Data were collected by the M.A.P.-RT module	A
Fang et al. (23)	Head and neck cancer	55	Patient and caregiver	A Web-Based Supportive Care Program	Study assessments	D
Kaimal et al. (24)	Various cancers	22	Patient and caregiver	2 single-session arts-based approaches	Questionnaires with PROMIS	A
Karlsson et al. (28)	Laryngeal cancer	50	Caregiver	Not mentioned	Questionnaires	C
Lancellotta et al. (21)	Gynecological cancer	30	Patient	HAPPY – Humanity Assurance Protocol in interventional radiotherapy (brachytherapy)	Conversation	D
Llewellyn et al. (25)	Breast cancer	13	Patient and caregiver	Not mentioned	Interviews	B
Phahlamohlaka et al. (26)	Prostate cancer	305	Patient and caregiver	Not mentioned	Interviews	B, C
Rades et al. (22)	Lung cancer	77	Patient	Not mentioned	Complete the distress thermometer of the National Comprehensive Cancer Network	A
Riedl et al. (11)	Various cancers	944	Patient and caregiver	Not mentioned	Questionnaires	A
Seol et al. (27)	Various cancers	26	Patient and caregiver	Not mentioned	Questionnaires form EORTC	B
Suvaal et al. (9)	Gynecological cancer	220	Patient and caregiver	SPARC (Sexual rehabilitation Program After Radiotherapy for gynecological Cancer)	Questionnaires include PROMs and concern EORTC	C

A: Psychological distress; B: Information provision; C: Sex and relations; D: Fear and pain.

For the subject number, 13 articles have less than 100 subjects (6, 12, 17–25, 27, 28). One paper has a much higher number of subjects than the others, including more than 1000 subjects (16). Nine papers have proposed interventions or programs for investigation (6, 9, 12, 16, 19–21, 23, 24). Inventions include a nurse-led aftercare program and a web-based supportive care program (12, 23). Notably, 7 papers use questionnaires as the measurement tool (9, 11, 16, 17, 24, 27, 28), among which 3 articles utilize questionnaires from The European Organization for Research and Treatment of Cancer (EORTC) (9, 17, 27). Lastly, 4 papers use interviews for the measurement (6, 18, 25, 26).

Four psychosocial needs were identified. The four needs were commonly reported in the selected papers, ensuring the generalizability. The indication of the key findings from each paper was listed in **Table 2**. Seven out of 17 papers mentioned or concluded that psychological distress was a common and important psychosocial concern (6, 11, 16, 17, 20, 22, 24). Five papers highlighted the need for improving information provision (18, 19, 25–27). Three papers pointed out the deterioration of social relationships as a psychosocial need (9, 26, 28). Two papers reported fear and pain as common psychosocial needs (21, 23). One paper covered 2 of the above four key findings (26), and one paper had no specific key finding (12). The four psychosocial needs were analyzed in the discussion. The solutions are also included in the discussion to offer future research directions.

### 3.3 Grading

To assess and compare the quality of the selected papers, the methodological index for non-randomized studies (MINORS) was used as the grading approach [12]. Some characteristics were found during the grading process. Firstly, all papers received the full mark of 2 in the aspects of inclusion of consecutive patients and appropriate endpoint to the study’s aim. In the part with a clearly stated purpose, the average score was 1.94, being the second-best performed category. The worst performance was in the area of unbiased assessment, which only had 0.059 out of 2 on average. The second lowest average score was regarding the prospective calculation of study size, which only had 0.235 out of 2. In total 13 papers were classified as “good” with a total score equal to or above 10. Among the 13 “good” papers, 8 of them had a total score of 12 which was the highest score. The remaining 4 articles were classified as “bad” with 8 to 9 scores. The average score was 10.65, which was above the benchmark of a “good” paper in the MINORS approach. **Table 3** shows the grading criteria and the performance of each selected paper.

**Table 3:** The grading of the 17 selected papers. MINORS is used as the grading approach.

Article	A clearly stated aim	Inclusion of consecutive patients	Prospective collection of data	Endpoints appropriate to the aim of the study	Unbiased assessment of the study endpoint	Follow up period appropriate to the aim of the study	Loss of follow up less than 5%	Prospective calculation of the study size	Score (/16)	Grading
Adeberg et al. (16)	2	2	2	2	0	0	0	0	8	Bad
Ahmadsei et al. (17)	2	2	2	2	0	2	2	0	12	Good
Andersen et al. (18)	2	2	2	2	0	2	2	0	12	Good
Ashmore et al. (19)	2	2	0	2	0	2	0	2	10	Good
Braat et al. (12)	2	2	0	2	0	1	2	0	9	Bad
Catherine et al. (6)	2	2	2	2	0	0	2	0	10	Good
Chiesa et al. (20)	2	2	2	2	0	2	2	0	12	Good
Fang et al. (23)	2	2	2	2	0	2	0	0	10	Good
Kaimal et al. (24)	1	2	2	2	1	0	0	0	8	Bad
Karlsson et al. (28)	2	2	2	2	0	2	2	0	12	Good
Lancellotta et al. (21)	2	2	2	2	0	0	2	0	10	Good
Llewellyn et al. (25)	2	2	2	2	0	0	2	2	12	Good
Phahlamohlaka et al. (26)	2	2	2	2	0	2	2	0	12	Good
Rades et al. (22)	2	2	2	2	0	1	2	0	11	Good
Riedl et al. (11)	2	2	2	2	0	1	0	0	9	Bad
Seol et al. (27)	2	2	2	2	0	2	2	0	12	Good
Suvaal et al. (9)	2	2	2	2	0	2	2	0	12	Good

Grading criteria include a clearly stated aim, inclusion of consecutive patients, prospective collection of data, endpoints appropriate to the aim of the study, unbiased assessment of the study endpoint, follow up period appropriate to the aim of the study, loss of follow up less than 5% and prospective calculation of the study size. The criteria refer to Greenhalgh’s study published in 1997 (14).

## 4 Discussion

**Figure 2:**
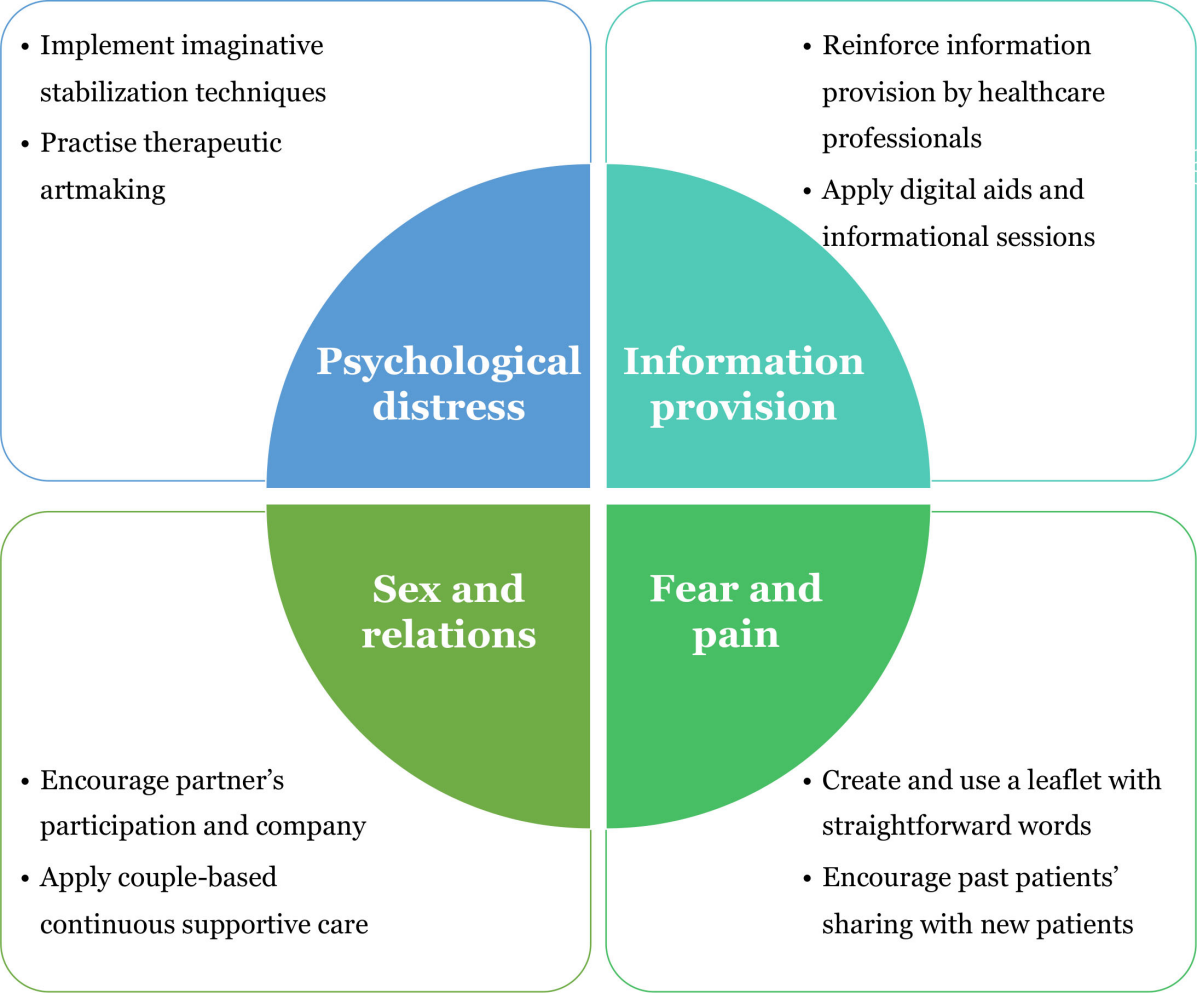
Summary of findings including the four psychosocial needs identified from the 17 selected papers and the proposed solutions for further study.

This systematic review aims to identify the psychosocial needs commonly encountered by post-radiotherapy cancer patients and their direct caregivers. Seventeen studies were selected for analysis and discussion. In this part, key findings are summarized into four needs. To offer research directions for future studies, interventions tackling each of the identified needs are provided. **Figure 2** summarizes the key findings and suggestions. The strengths and weaknesses of this review are investigated with suggestions for future studies in the final section.

### 4.1 Key findings

#### 4.1.1 Psychological distress

The first key finding is the need to address the psychological distress of cancer patients due to radiotherapy. According to the study by Ahmadsei et al. (17), about half of patients reported increased psychological distress during and after multiple sessions of radiotherapy. Another study used the Hospital Anxiety and Depression Scale to indicate the presence of affective psychopathology in radiotherapy patients (6). The patient’s quality of life was worsened due to the radiotherapy-derived psychological issues. Psychological distress can be derived from treatment procedures. Mask fixation is a procedure of wearing and fitting the radiotherapy mask on the patient’s head region for immobilization. A study showed that patients experienced wearing a mask as distressing, or even suffered from “mask anxiety” (16). Psychological distress can also be caused by physical issues. For instance, sleep disturbances were proven to be associated with the high distress score of 77 lung cancer patients (22), further leading to emotional problems and a request for psychological support.

Besides cancer patients, their caregivers also suffer from psychological distress. A study showed that family members of cancer patients were distressed and warranted additional support (11). From the same study, 17.2% of patients reported that their illness affected family members psychologically.

Some of the selected papers suggested interventions to relieve patients’ psychological distress. For instance, an intervention named imaginative stabilization techniques was suggested to alleviate patients’ psychological distress by increasing relaxation for patients undergoing mask fixation and radiotherapy (16). The techniques assisted patients in gaining control over the overwhelming feelings in the treatment process.

Besides, there are other suggestions for alleviating patients’ psychological distress. Therapeutic artmaking could support cancer patients’ emotional and psychological needs caused by radiotherapy (24). Open studio art therapy refers to a dedicated studio space with a range of artistic and expressive opportunities. Open studio art therapy could improve the well-being of patients undergoing stressors of oncology treatment. The therapeutic intervention was highly recommended to relieve patients’ psychological tension and improve their quality of life. Additionally, combining psychological interventions with standard therapies could reduce the need for sedation, further improving patients’ well-being and treatment experience (20). Further research is required to validate the effectiveness and feasibility of the pilot interventions.

### 4.1.2 Information provision

Secondly, there is a need for improving information provision. It was common for patients to have a sense of “unknown” due to the lack of information (21). The study stated that not all the information given to the patients could be understood at the first time. Notably, worry due to a low level of understanding was associated with anxiety and depression. Therefore, it is important to ensure and strengthen information provision.

Sufficient information was necessary to reduce uncertainty and guarantee feelings of safety and predictability (18). In the study, skin reactions from radiotherapy were used as an example to show the paramount importance of adequate information provision. The primary source of information such as oral and written information provided by the oncology department was considered trustworthy to patients. It was highlighted that patients preferred references from medical professionals to Internet resources. General information and advice from physicians, radiation therapists or nurses to confirm if reactions were normal or not were important to patients in aftercare. Therefore, the provision of information regarding common post-radiotherapy symptoms by healthcare professionals is strongly suggested.

Another study reported cancer patients’ overwhelming wish for an instant access to reliable and relevant information in one place (19). Better information on late side effects and discussion on sexual health were required by the patients. Other than physical information delivery such as leaflets, a digital information platform was viewed as a possible intervention which reassured patients.

There are studies other than the 17 selected papers supporting the importance of information provision. It was proven that information provision was one of the most common forms of education for pediatric patients and their families (29). There were various suggestions for information delivery including audio and visual aids, teaching dolls and informational pamphlets. Facility tours and informational sessions were suggested to be provided to enhance patients’ and caregivers’ understanding of the treatment. In terms of social needs, financial difficulty was significantly associated with a low quality of life score of patients after radiotherapy (27). The related practical information provided by healthcare professionals or medical social workers may help patients and their caregivers to alleviate the mental burden, satisfying their psychosocial needs and improving the comprehensiveness of healthcare service.

#### 4.1.3 Sex and relations

Social needs are derived after radiotherapy. Affected sexual functions lead to a worsened social relationship, being a significant social need. In a study of 22 women with gynecological cancer treated with radiotherapy, an improvement in the care of sexual function and relationship management was suggested (9). Approximately one-third of gynecological cancer patients including cervical, uterine and vaginal cancers received radiotherapy. Sexual problems including dyspareunia and vaginal dryness were frequently reported. Eventually, relationship dissatisfaction was likely to occur, deteriorating patients’ relationships and social well-being.

Another study showed the loss of sexual function in prostate cancer patients after radiotherapy (26). The study pointed out that the affected sexual function had a detrimental impact on men’s quality of life, psychological well-being and intimate relationships. Prostate cancer patients reported failure at achieving intimacy with their partners due to affected sexual functions. Therefore, it is vital to improve the post-treatment care of sexual function and relationship management.

Besides cancer patients, their caregivers also suffer from relationship problems. In a study that included 50 caregivers of patients with laryngeal cancer treated by radiotherapy, impacts on relationships with patients and others were reported (28). Caregivers such as partners suffered from a loss of intimacy due to the patient’s physical illnesses (26). Notably, the relationship problems affected male more than female caregivers. Caregivers of late-stage patients were more socially affected than caregivers of early-stage patients.

To tackle the relationship problems of patients and caregivers, a study proposed a nurse-led sexual rehabilitation which reinforced the partner’s participation and accompanying the patients (9). Face-to-face sessions were provided to the couples on related topics such as fear of resuming sexual activity after cancer and mutual coping promotion. The suggested nurse-led intervention could help patients and their partners to obtain mutual understanding. When both sides of a couple received professional information and guidance, they were likely to have less dissatisfaction with intimacy and relationship. Also, continuous care provided by the radiotherapy department was recommended and required by patients (26). The patients should be given opportunities and time to discuss their experiences with healthcare providers in a quiet and private environment. It may help patients in need to better recover from their affected sexuality and relationship.

A recent study stated that “relationships change before, during and after cancer treatment” (30), echoing the importance of continuous supportive care. The above study revealed that partnerships were changed into patient-and-caregiver relationships. The change of role led to relationship distress and increased depression in both patients and caregivers. To tackle the relationship concern, a couple-based intervention was suggested (31). In the study, 43 pairs of couples were recruited and provided with psychosexual intervention. The intervention was proven to be feasible and acceptable. Most importantly, the couple-based program led to a decrease in anxiety and depression. All these show that partners’ participation carries the utmost importance to repair a relationship and tackle patients’ and caregivers’ psychosocial needs.

#### 4.1.4 Fear and pain

Fear and pain are common findings from the selected papers. In a study assessing the needs of 30 gynecological cancer patients, not knowing what to expect and the fear of feeling pain were found to be the significant sources of concern for 76.7% of the patients (21). For the lack of understanding, it was closely related to the words that patients listened to during their treatment journeys. The same study showed that 33.3% of patients felt a sense of insecurity from the word “brachytherapy” as it was usually a new and strange word to them. Meanwhile, it was reported that “interventional radiotherapy” sounded more reassuring because it was more familiar and likely to refer to a minimally invasive procedure. Additionally, all assessed patients did not appreciate the use of the word “bunker” which described the treatment place. Besides, the fear of feeling pain during treatment procedures was also a major concern for patients (21). The paper suggested that pain was closely associated with anxiety or depression scores. All these show that fear of pain and uncertainty is a common psychosocial need that requires extra attention and care.

Patients’ fear and pain were also reported in a web-based program which provided information about managing symptom-focused concerns (23). Post-radiotherapy patients of oral cancers were invited to use and evaluate the program. 70% of the participants visited the program more than once. Notably, the participants spent most of the time viewing the unit about coping with pain. It shows that pain is a common concern for post-radiotherapy patients.

Suggestions are provided to alleviate patients’ fear and tackle their psychosocial needs. First, it is recommended to pay more attention to the choice of words. More straightforward and familiar words should be used during communication between healthcare professionals and patients. For instance, a study suggested using an “interventional room” or “treatment room” to replace a “bunker”, which was less familiar to most patients (21). Also, more conventional terms such as “interventional radiotherapy” could be used to refer to “brachytherapy” to reassure patients. The same study recommended the creation and use of a procedure information booklet. Treatment information and past patients’ stories could also be shared with new patients through the booklet distributed before the treatment. Remarkably, the use of decision support tools and predictive models was highlighted to minimize patients’ fear of the “unknown”.

There are different sources of fear. From a supporting study, the subjects had fears of relying on caregivers (32). The fear of dependency was one of the reasons leading to suicidal thoughts, worsening the patient’s psychological well-being. Pain is often originated from physical complications due to radiation. For instance, patients with head and neck cancer might need radical dental treatment such as prophylactic tooth extraction, which caused pain in chewing and speaking (15). The same study also pointed out that patients commonly reported fear such as the fear of cancer recurrence. All these show that fear and pain are significant psychosocial needs of post-radiotherapy cancer patients.

### 4.2 Future research

Firstly, more studies on the psychosocial needs of caregivers of radiotherapy patients are highly suggested. Among the 17 articles selected, patients are usually the primary target of the study. Only 1 article explicitly studies the needs of caregivers although their needs are discussed in other studies together with the patient’s needs (28). Caregivers of patients undergoing radiotherapy are likely to encounter mental issues. A study on laryngeal cancer radiotherapy caregivers showed that up to 38% of caregivers reported psychiatric disorders (28). Tremendous demands and burdens are placed on the caregivers’ shoulders during care provision. Notably, caregivers often report negligence of health and a higher level of fear of cancer recurrence than the patients themselves. Socially speaking, caregivers may suffer from unemployment and relationship problems with others. All these prove that the psychosocial needs of caregivers are critical, and show the importance of future studies on the needs of caregivers.

Second, it is suggested to have further studies on diversified cancer types. In this study, the chosen articles commonly investigate either breast cancer or head and neck cancer (6, 12, 16, 18, 23, 25). However, for lung cancer, there is only one related study found (22). For other common cancers including liver and colorectal cancer, the number of studies on psychosocial needs is insufficient. It is difficult to understand and identify cancer-specific psychological and social concerns if possible. Therefore, it is recommended to have future research on different cancers and the specific psychosocial needs encountered by patients and caregivers. More studies should be conducted on cancers such as colorectal and prostate cancer which are less studied. For example, Loi et al. (33) conducted a systematic review on post-radiotherapy sexual health difference between stereotactic body radiotherapy (SBRT) and conventional regimens in prostate cancer patients. Although this systematic review could only demonstrate similar decline in sexual function after SBRT versus conventional regimen, this sheds the light that within a specific cancer type, radiotherapy technique, fractionation, and specific psychosocial item could be investigated for suggesting improvements in post-radiotherapy psychosocial health. For the cancers which have been widely investigated, the caregivers’ needs can be the next research target. There is a significant proportion of papers focusing on a range of cancers treated by radiotherapy rather than a specific cancer type. Therefore, it is suggested to conduct future studies on cancer-specific psychological and social concerns.

Thirdly, it is suggested to enhance studies’ fairness and creditability. MINORS is used to assess the quality of the papers. All papers receive the full mark of 2 in the areas of inclusion of consecutive patients and appropriate endpoint to the study’s aim. It shows that all papers can investigate eligible subjects and conduct research within a well-stated period. Also, the area of a clearly stated aim has the second highest average score of 1.94 out of 2. It highlights the importance of stating a study’s aim explicitly in an article. The worst performance is in the area of unbiased assessment. The subjects are usually patients with radiotherapy or caregivers, and they are not sufficiently randomized. “Blind” assessment is not common in articles in this field. It may be due to the increased cost and difficulty. For instance, for an interview study of post-therapy patients, doing a “blind” test will not be possible or desirable. The second lowest average is about the study size. Among the 17 selected articles, only 4 have more than 100 subjects included in their research. The number of subjects ranges from 7 to 1020. The subject quantity should be statistically significant to support creditable findings. Therefore, it is recommended to increase the subject size and fairness. For instance, more subjects can be recruited through various channels. All eligible subjects should be invited in order to minimize the selection bias. Also, well-constructed scales such as the Hospital Anxiety and Depression Scale can be applied to assess psychopathology (6). It can help identify psychological concerns logically. Randomized controlled trials, such as comparing groups with or within radiotherapy, are currently not common due to the difficulty but have the value of study. It is recommended to conduct randomized controlled trials to study the effectiveness of the suggested interventions. By comparing the psychological well-being with or without an additional intervention, the effect of the intervention on post-radiotherapy patients or caregivers can be investigated.

### 4.3 Strengths and weaknesses

To assess the quality of this study, the first strength is the thorough and diversified literature review process. Advanced search is used in various massive and reputable online databases, including PubMed, Embase and Scopus. The widely accepted PRISMA model is applied to construct an organized selection process (13). The model also facilitates understanding the common reason for exclusion, such as not focusing on radiotherapy or being unrelated to psychosocial needs. It assists the literature research process of future studies.

The second strength of this study is the analysis of high-quality papers. MINORS is used as the grading approach. From the quality assessment, 13 papers are classified as “good” and the average score is above 10, showing the high quality of the papers selected for this review.

Nevertheless, this systematic review has a few limitations. The main limitation is the limited number of papers selected for discussion. It implies that limited studies have been done in the field related to post-radiotherapy psychosocial needs. This review can provide information to encourage future studies in the related fields. Furthermore, the diversified results from different papers lead to limited common findings. Despite the selected papers being generally considered as high quality, the performance in the area of unbiased assessment and study size calculation falls short of the standard, lowering the level of evidence. Lastly, it is acknowledged that psychosocial needs can be caused by multiple factors other than radiotherapy, such as the announcement of a cancer diagnosis. It is also acknowledged that psychosocial needs are subject to cancer sites and treatment modalities. This review aims to identify the needs encountered by post-radiotherapy patients and caregivers. Future studies are encouraged to investigate the causes and related interventions.

## 5 Conclusions

Post-radiotherapy patients and caregivers encounter different psychosocial needs subject to various reasons. Radiotherapy is an indispensable cancer treatment modality. Meanwhile, the derived psychosocial needs should not be left unmet, worsening patients’ and caregivers’ quality of life. Some interventions cater to the identified needs and can be actualized by related healthcare professionals. More future studies on the needs of patients and their caregivers are highly encouraged. Applying the creative interventions warrants more studies in both research and clinical fields.

## Data availability statement

The original contributions presented in the study are included in the article/supplementary material. Further inquiries can be directed to the corresponding authors.

## Author contributions

Conceptualization, SYT; writing—original draft preparation, KHM; writing—review and editing, SYT and HK-WL.; supervision, SYT. All authors contributed to manuscript revision, read, and approved the submitted version.
